# Descending Colon Leiomyoma: A Case Report and Literature Review

**DOI:** 10.7759/cureus.67390

**Published:** 2024-08-21

**Authors:** Norah I Alabdulaaly, Bader D Alanazi, Khalid A Albassam, Nada N Binkhashlan, Saad T Alqahtani, Nahla S Arab, Fatima A Badahdah, Saeed S Albalawi

**Affiliations:** 1 General Surgery, Prince Sultan Military Medical City, Riyadh, SAU; 2 General Surgery, Prince Sultan Military Medical City, Riyadh , SAU

**Keywords:** gastrointestinal tract, smooth muscle tumors, descending colon mass, large bowel, leiomyoma

## Abstract

Leiomyoma is defined as a benign proliferation of smooth muscle cells. Smooth muscle tumors are considered the second-most common mesenchymal neoplasm in the gastrointestinal (GI) tract. It typically occurs incidentally in the large bowel. Colonic leiomyomas are considered to be very rare and commonly found in the descending or sigmoid colon. We report a case of a 32-year-old woman with no previous medical illness who presented with on-and-off abdominal pain, was found to have a left colonic mass, and underwent laparoscopic left hemicolectomy, with the final histopathological assessment revealing smooth muscle leiomyoma.

## Introduction

The definition of colonic leiomyoma is a benign proliferation of smooth muscle cells. They are usually found incidentally on colonoscopy or radiological imaging [1]. The most common site for gastrointestinal (GI) leiomyomas is found in the esophagus or stomach [1]. Colonic leiomyomas represent only 3% of all gastrointestinal leiomyomas; thus, they are considered extraordinarily rare [2]. From the whole colon, the descending and sigmoid colon are the most common sites for developing leiomyomas, usually as intraluminal polyps [3]. In this case, we reported a large colonic leiomyoma involving the descending colon.

## Case presentation

A 32-year-old female patient presented to the general surgery clinic with a three-month history of recurrent attacks of left lower quadrant (LLQ) abdominal pain. The pain was colicky in nature, intermittent, gradually appearing and progressing slowly, lasting for 30 minutes, on and off. The pain was associated with nausea, however, without vomiting, and it was also associated with per rectum (PR) bleeding (small amount, fresh blood, no clots). The pain was aggravated by constipation and relieved by analgesia. The patient denied any history of fever, chills, weight loss, urinary symptoms, or diarrhea. Her past medical history was not significant for any chronic medical illness, and her surgical history was unremarkable. Her family history was negative for malignancy. 

On presentation to the clinic, the patient was vitally stable. She was sitting comfortably, not in pain or distress, and had a well-built body. Her abdominal examination showed no previous surgical scars and was soft, with no tenderness or palpable mass in the LLQ. The mass was smooth with a regular edge, about 7 cm × 8 cm, and non-mobile with no skin changes. The PR examination showed a good anal tone with no bleeding or palpable masses.

The patient underwent a blood workup, including tumor markers. The tests showed an alpha-fetoprotein test of 0.8 ng/mL, cancer antigen 19-9 of 13 ng/mL, and carcinoembryonic antigen of 0.9 ng/mL. However, the results of other laboratory examinations were unremarkable. The patient underwent a colonoscopy, which showed a fungating ulcerating mass in the colon about 50 cm from the anal verge, at which the scope could not advance beyond it. Multiple biopsies were taken from the mass. The biopsy came back as smooth muscle neoplasm in favor of leiomyoma. The patient was further investigated with a computed tomography (CT) scan of the abdomen and chest for staging.

**Figure 1:**
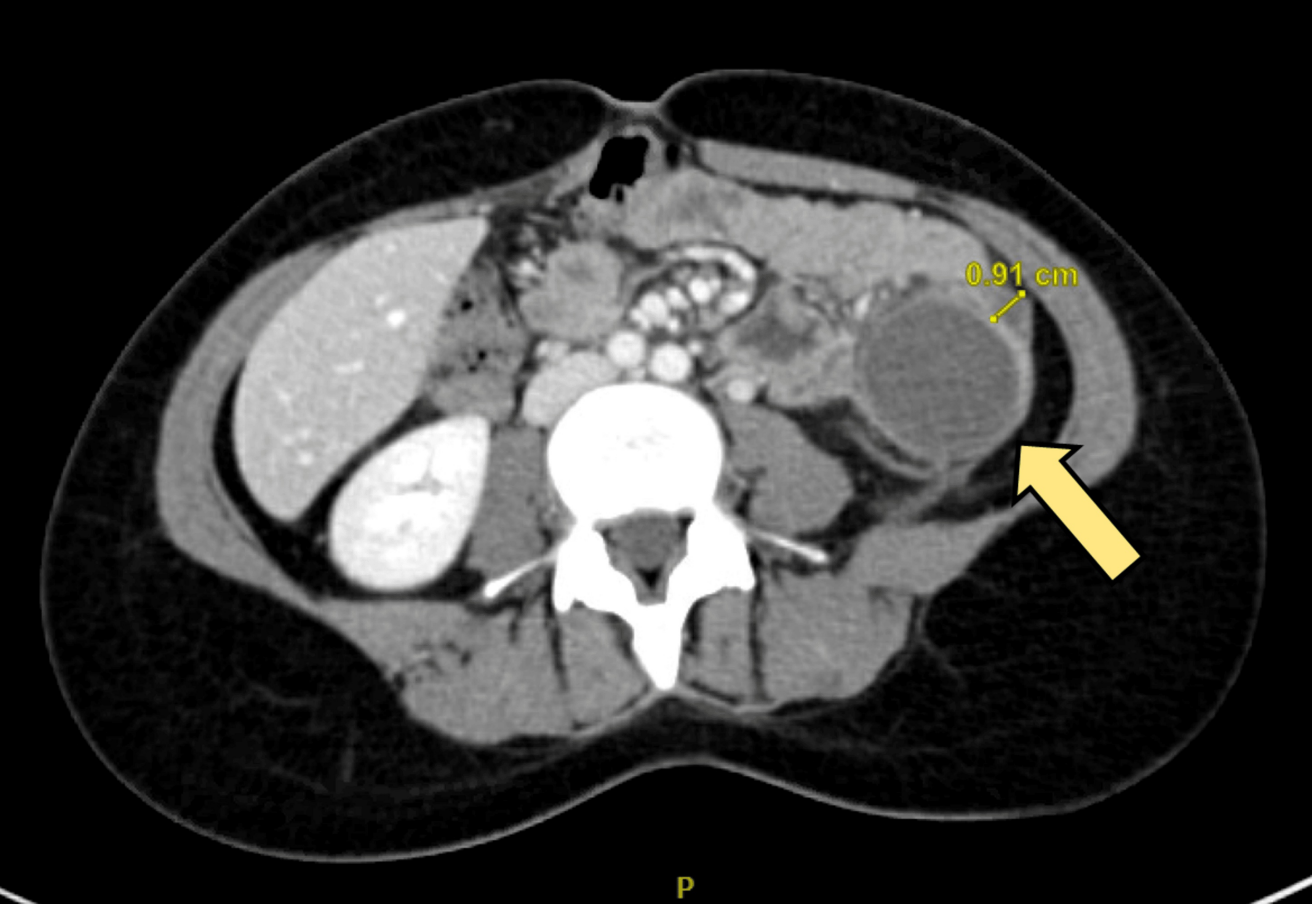
Axial view CT scan showing left sided colonic mass The yellow arrow is indicating the left-sided colonic mass. The 0.91 cm measurement indicates the thickness of the wall of the mass.

**Figure 2:**
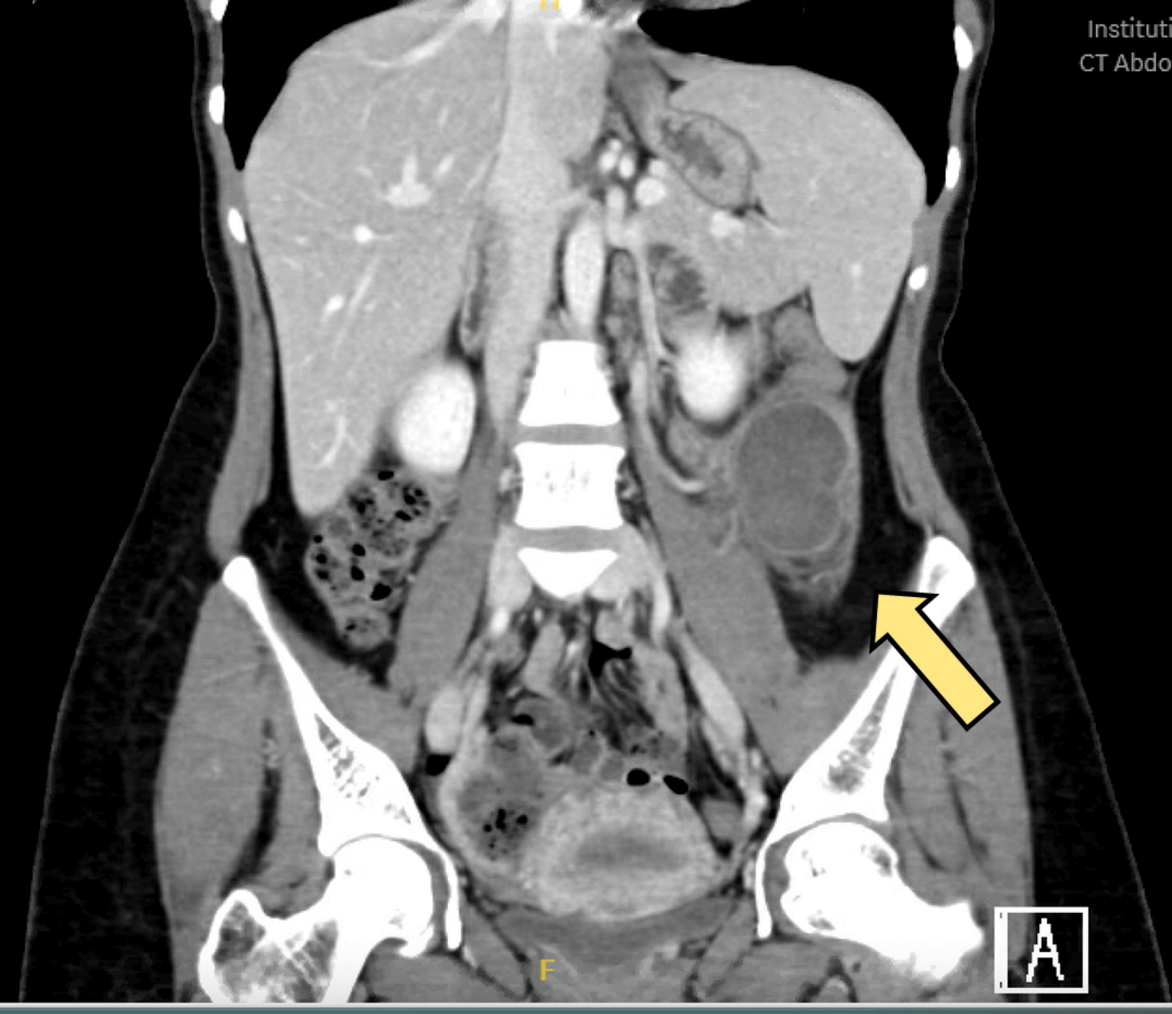
Coronal view CT scan showing left sided colonic mass The yellow arrow indicates the left-sided colonic mass.

**Figure 3:**
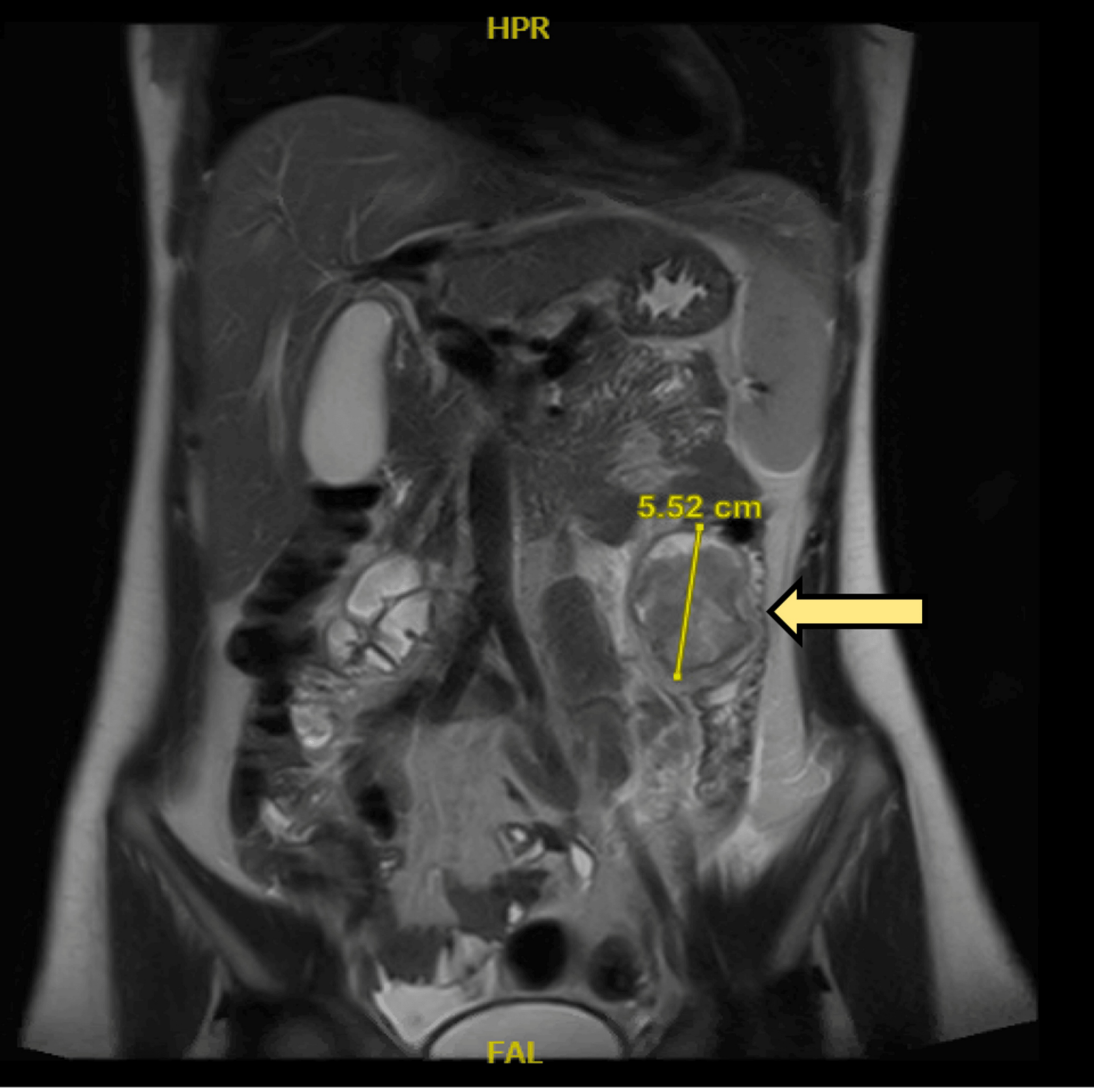
Axial view of magnetic resonance imaging showing left-sided colonic mass The yellow arrow is indicating the mass (the mass is measured 5.52 cm).

**Figure 4:**
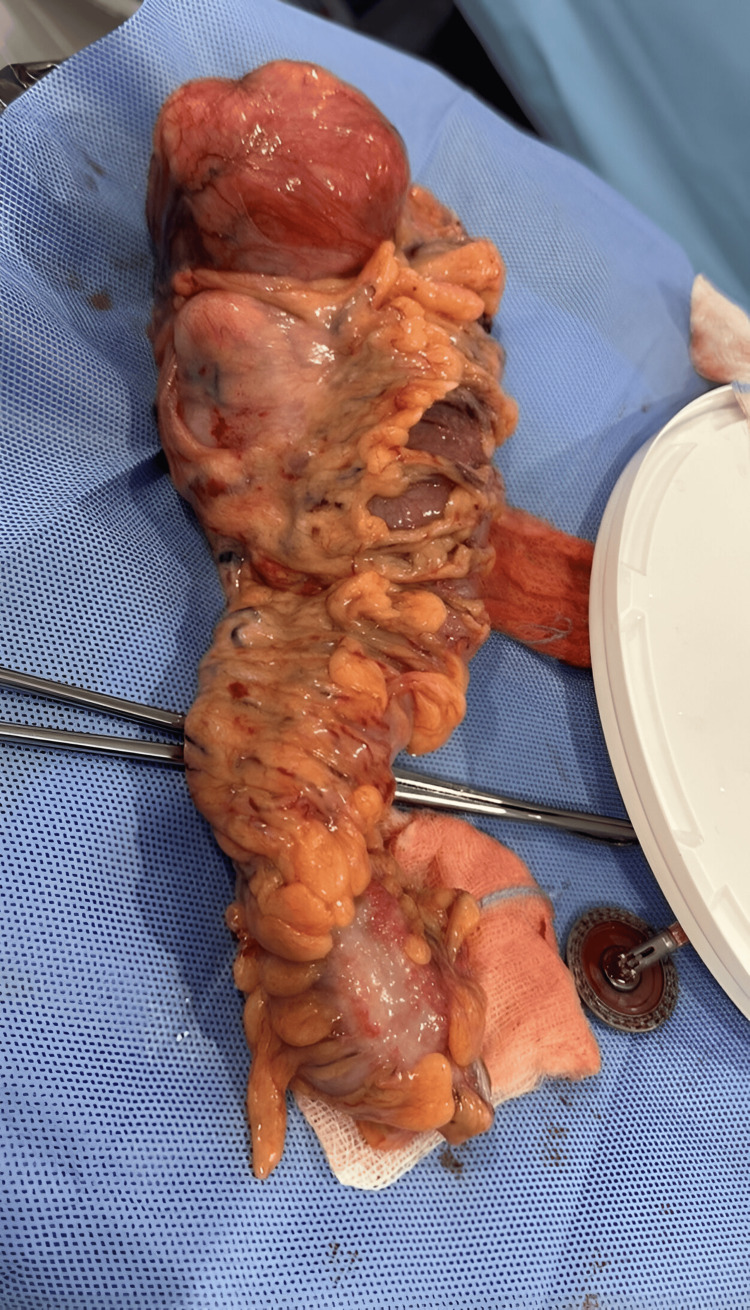
Intra-operative finding of resected descending colon mass

A CT scan with intravenous contrast of the abdomen showed a descending colon intraluminal mass with a huge external mural component extending into the mesentery, collectively measuring 8 cm × 11 cm. The mass is inseparable from the proximal jejunal loops without definite signs of invasion. There were no signs of obstruction. There is no synchronous lesion. There was no evidence of abdominopelvic lymphadenopathy or metastasis (T3N0), as shown in Figures 1-2. A CT of the chest showed no evidence of metastasis. Magnetic resonance imaging (MRI) of the abdomen was done for better mass characterization, which showed the same finding in the previously mentioned CT scan (Figure 3). The patient was planned for elective admission and underwent laparoscopic left hemicolectomy with primary anastomosis. The intra-operative finding of descending colon mass (Figure 4). The patient had an unremarkable post-operative course, started on a diet gradually, and started to pass bowel motion on the third day post-operatively. She was discharged home in her usual state of health on day 5 post-operatively, with follow-up in the clinic.

**Figure 5:**
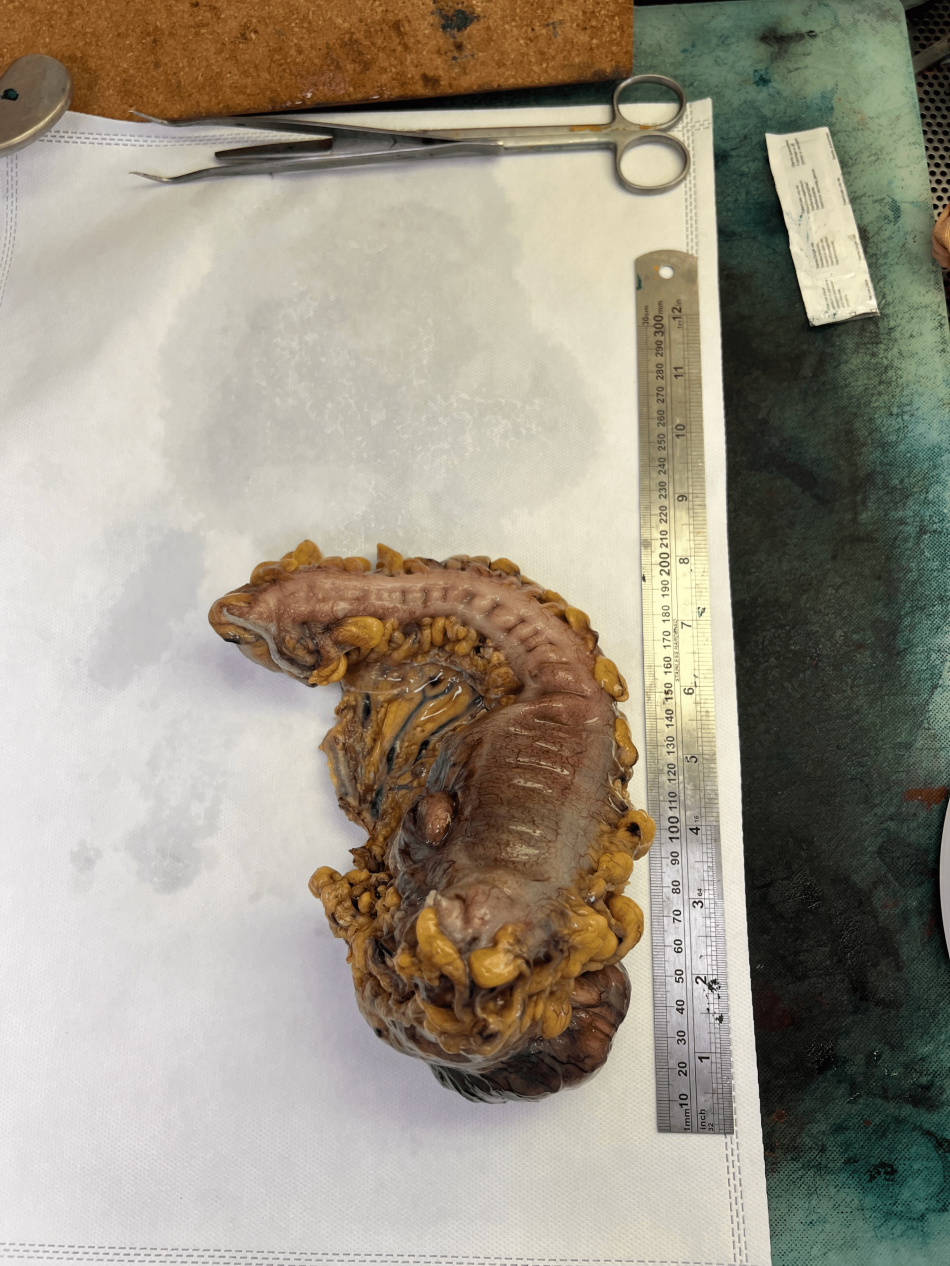
Intra-operative measurement of the resected mass The resected left colon measures about 20 cm. The tumor measures about 9 cm.

**Figure 6:**
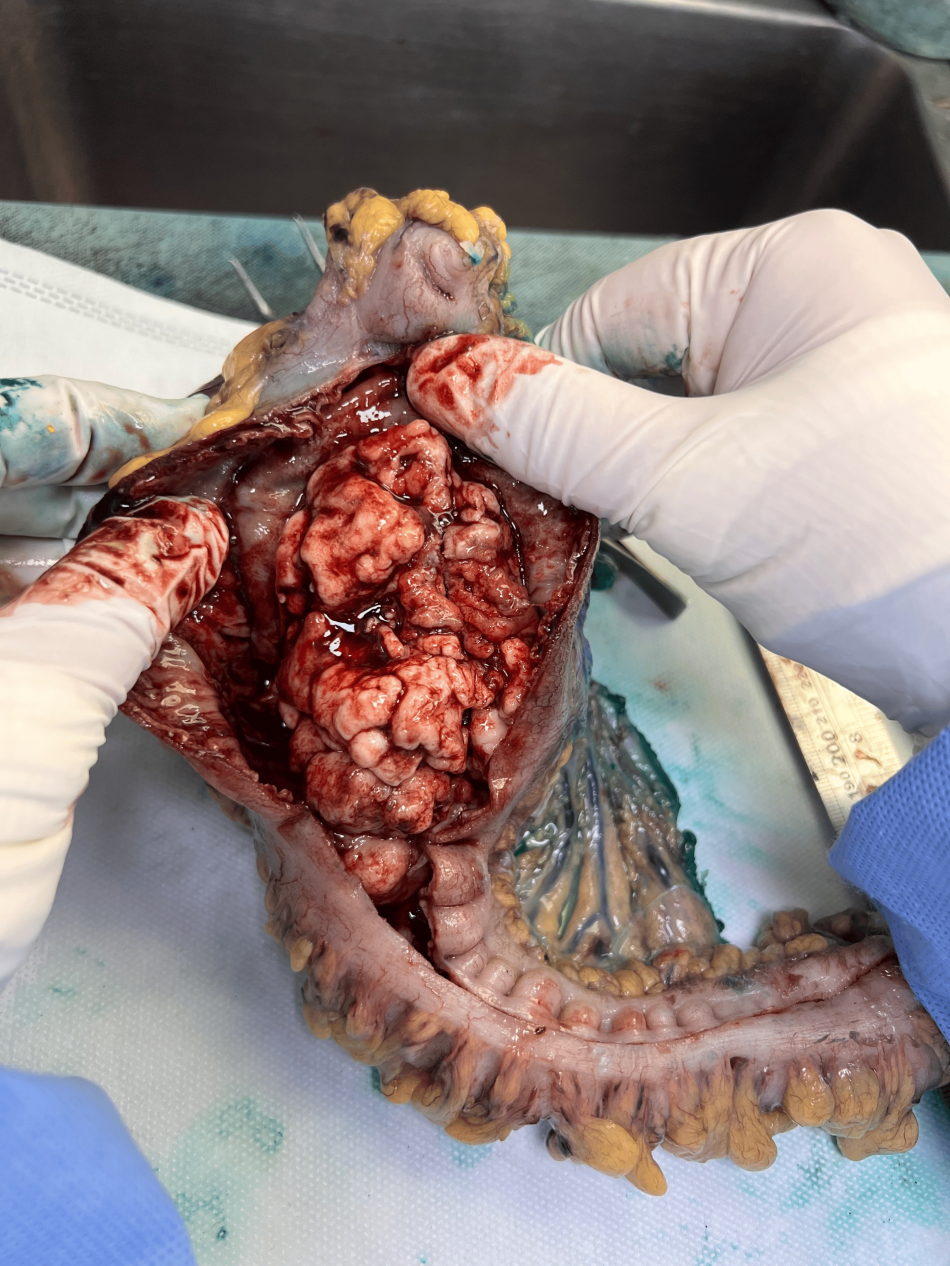
Intra-operative intra-luminal exploration of the mass

**Figure 7:**
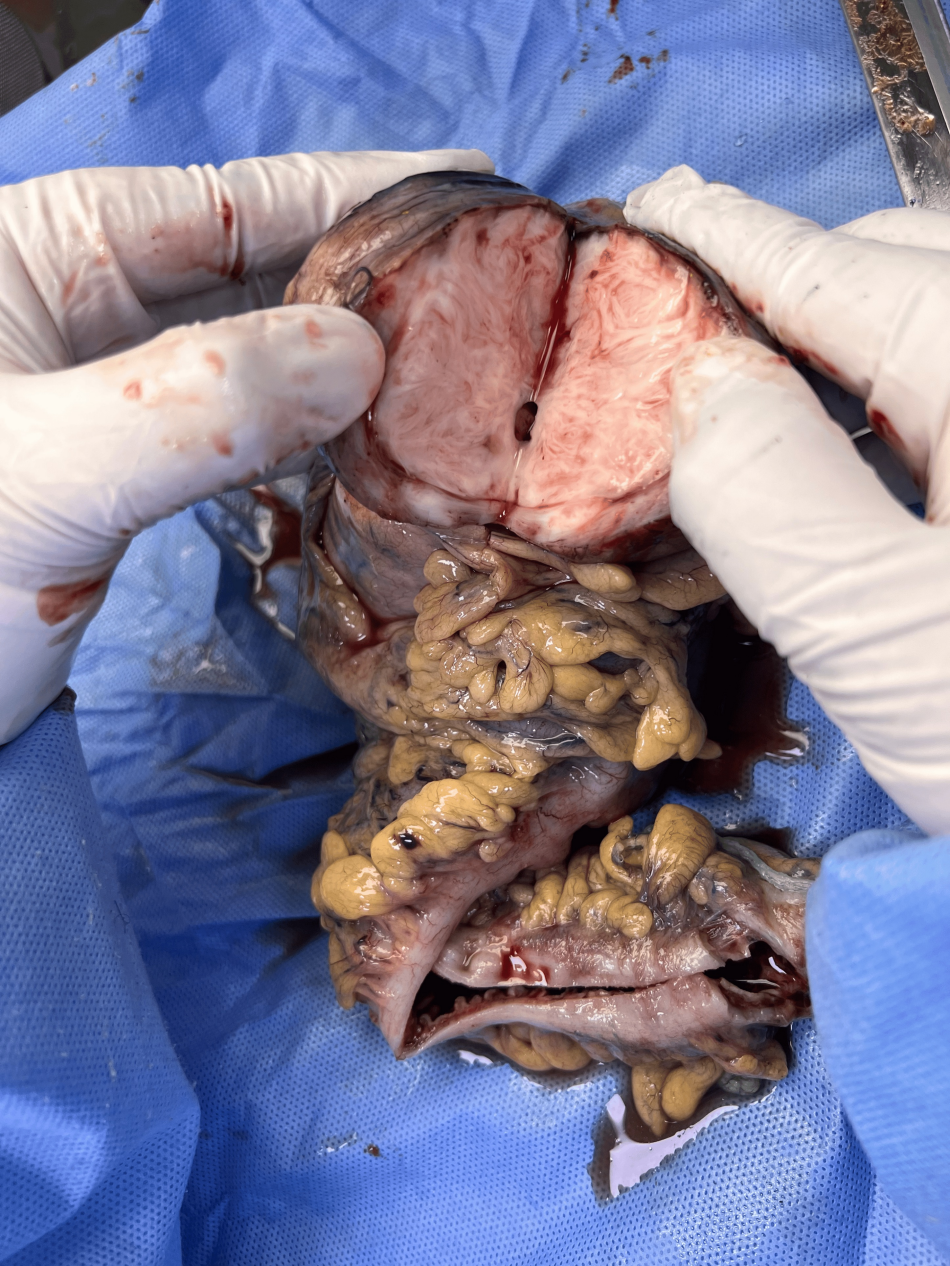
Intra-operative gross exploration of the mass showing the exophytic component of the mass

Histopathology examination of the specimen confirmed smooth muscle leiomyoma, a tumor size of 11 cm with innovation to the lamina propria, and mitotic activity of four mitoses per 5 mm^2^. All tumor margins were negative, with 16 negative lymph nodes (no evidence of malignancy) (Figures 5-7). The decision was made to discuss the case in the multidisciplinary meeting of the tumor board with a final plan for the oncology team review in the clinic for the decision. The patient was referred and seen in the medical oncology clinic with a plan for observation and follow-up clinically and radiologically, with no indication for chemotherapy. The patient kept on follow-up in the clinic with no evidence of recurrence noticed in the four-month period and planned to continue follow-up with clinical and radiological images.

## Discussion

The definition of colonic leiomyoma is a benign proliferation of smooth muscle cells. The esophagus, or stomach, is the most common site for GI leiomyomas [1]. This is in contrast to our patient with leiomyoma in the descending colon.

A study by Miettinen et al. found that these tumors have males predominating and a median age of 62 years [1]. In histological examination of these lesions, they are positive for smooth muscle actin and desmin but negative for CD34, CD117, or S100 protein [1].

The colonic leiomyomas are rarely presented with clinical signs and symptoms and are usually found incidentally on routine colonoscopy screening. However, just like our patient, they can present with a wide variety of symptoms, like abdominal pain, constipation, bleeding, or bowel obstruction if it is large [4]. Large leiomyomas (more than 2 cm) commonly lead to serious symptoms like altered bowel habits, a palpable abdominal mass, and/or bloody stools and may lead to bowel obstruction [5]. Rectal leiomyomas can manifest with melena or anal bleeding [6]. In women, rectal and sigmoid leiomyomas can present as a solid adnexal mass [7].

In general, colonic leiomyomas carry the risk of malignant transformation. High-risk tumors, including those with a high mitotic count, cellular atypia, and a large size of more than 2 cm, should raise suspicion for possible metastasis [4]. Leiomyosarcomas differ from leiomyomas in that they have significant atypia, are larger in size, are more aggressive, have a high mitoses rate, and carry a significant risk for malignancy. Radiological imaging and preoperative biopsy can be non-specific, and usually, a combined endoscopy and imaging studies such as CT scans and MRIs are required to establish the diagnosis [3]. On endoscopy, colonic leiomyomas appear as mucosal adenomas, and a biopsy and histological examination are required to establish and confirm the diagnosis [4]. The usual endoscopic appearance of colonic leiomyomas appears as firm, well-circumscribed, intraluminal, or pedunculated polyps [8]. Due to this similarity in the appearance of mucosal polyps and colonic leiomyomas, misdiagnosis may occur, with only about 46% of colorectal leiomyomas being diagnosed accurately based on their endoscopic features, as reported by Choi et al. [5]. However, there is an increased rate of accurate diagnosis of colorectal leiomyoma as more frequent screening colonoscopies are performed [5].

The best treatment method for colonic leiomyomas, in general, is surgical excision. However, operative management has a wide variety, from simple endoscopic excision to subtotal colectomy based on the size of the leiomyoma [4]. Most of the patients eventually undergo surgical excision to achieve complete removal. However, the management plan is determined based on histological findings, location, tumor size, and mitotic count [3]. Usually, it is difficult to differentiate between benign and malignant tumors perioperatively; the most appropriate recommended treatment is wide resection for smooth muscle tumors of the GI tract [4]. Colonoscopic removal is preserved for small polyps, and surgical resection should be considered for large pedunculated or sessile leiomyomas [4]. There are a few case reports of endoscopic removal of leiomyoma of the colon, and all the cases involved a small (less than 2 cm in diameter) leiomyoma [2].

The most important prognostic factor for colorectal smooth-muscle tumors, in general, is the tumor size [8]. A tumor size of more than 5 cm is highly suggestive of malignancy [5]. In general, the prognosis of colonic leiomyoma is good, with no reported recurrence after resection in the literature [3]. Surgical resection is preferred in cases of large tumors (more than 5 cm) or in cases where there is a feature worrisome for malignancy [5,9]. A complete wedge colonic resection is required for larger lesions to achieve complete removal [3].

## Conclusions

Colonic leiomyomas are considered a rare type of GI tumor and are usually found incidentally during screening colonoscopy. Diagnosing these leiomyomas usually requires combined radiological, colonoscopic, and histopathological analysis to establish the diagnosis. Surgical excision is the treatment of choice for large and highly malignant potential colonic leiomyomas. In general, it has a good prognosis after the resection, with no evidence of recurrence reported in the literature. Clinical and radiological follow-up is recommended after surgical resection for large leiomyomas.
